# ClinVar Database Evolution and Impact on Potential Pathogenic Germline Variant Reporting from Tumor Comprehensive Genomic Profiling

**DOI:** 10.1158/2767-9764.CRC-25-0038

**Published:** 2025-08-05

**Authors:** Allison W. Kurian, Erica Gornstein, Lisa Heppler, Kali Chatham Dougherty, Rachel B. Keller-Evans

**Affiliations:** 1 Stanford University School Of Medicine, Stanford, California.; 2 Foundation Medicine, Inc., Cambridge, Massachusetts.

## Abstract

Although recommended for a high percentage of patients with cancer, germline genetic testing is widely underutilized. Classification of pathogenic variants of potential germline origin (potential pathogenic germline variants; PPGV) from tumor comprehensive genomic profiling enables identification of patients and families with high cancer risk via an alternative pathway to clinical germline evaluation. Foundation Medicine utilizes evidence in the ClinVar database to classify PPGVs. We investigated the impact of ClinVar database evolution over a 2-year period on the classification of PPGVs across solid tumors. Substantial growth in the database (a 52.2% increase in classified variants across 24 cancer susceptibility genes) yielded a modest increase in PPGV prevalence (+0.5% across tissue and liquid biopsies combined). Whereas filtering of PPGVs because of insufficient evidence for pathogenic classification decreased across all ancestry groups, disproportionate filtering of variants in patients of South Asian (40.0%), admixed American (36.5%), and African (36.3%) versus European genomic ancestry (33.8%, *P* ≤ 0.01 for all comparisons) was still observed. Continuing improvements to genetic testing access and data sharing are needed to mitigate disparities.

**Significance:**

Whereas efforts to improve data sharing have led to the growth of public genomic databases (e.g., ClinVar) over time, underutilization of genetic testing and persistent racial and ethnic disparities are limitations that continue to affect these important resources.

## Introduction

Tumor comprehensive genomic profiling (CGP) is increasingly routine in clinical oncology practice and can lead to the identification of pathogenic variants of potential germline origin (potential pathogenic germline variants; PPGV). Whereas germline genetic testing is recommended for a large proportion of oncology patients, population-based studies have shown that it is frequently underutilized (1). Thus, reporting PPGVs that warrant confirmation through germline testing is a public health imperative.

We previously described the implementation of a germline banner feature on Foundation Medicine CGP reports which calls attention to PPGVs in 24 cancer susceptibility genes (2). Germline banner reporting relies on the ClinVar (3) genomic database for PPGV classification. However, ClinVar and other existing public genomic databases are limited by historically unequal representation of racial and ethnic ancestries in both clinical studies and commercial genetic testing. Prior large-scale studies have found an excess of variants of uncertain significance (VUS) in historically underserved/undertested populations (1, 4), and we previously noted disproportionate filtering of PPGVs across genomic ancestries in a cohort of patients who underwent Foundation Medicine CGP (2). This disparity has the potential to widen gaps in the quality of cancer care.

In 2023, a major germline testing laboratory initiated sharing of variant classification data into ClinVar, thus making these data publicly available. We investigated the effect of evolution of the ClinVar database over time, including expansion during the estimated time period of this large-volume data share, on germline banner reporting of PPGVs across cancer susceptibility genes and cancer types and evaluated whether evolution of the database has reduced ancestral bias affecting PPGV classification.

## Materials and Methods

### Study cohort

We queried an institutional database (Foundation Medicine, Inc.) to identify the study cohort (*N* = 289,547) which consisted of solid tumor tissue biopsy (*N* = 222,241) and liquid biopsy (*N* = 67,306) CGP results reported during routine clinical care between January 2021—following implementation of the germline banner on Foundation Medicine reports—and July 2024 (Supplementary Table S1). Approval for this study, including a waiver of informed consent and a Health Insurance Portability and Accountability Act waiver of authorization, was obtained from the Western Institutional Review Board (protocol number 20152817).

### CGP

Hybrid capture–based next-generation sequencing assays were performed on patient samples in a Clinical Laboratory Improvement Amendments–certified, College of American Pathologists–accredited, New York State–approved laboratory (Foundation Medicine, Inc.). FoundationOne CDx and FoundationOneLiquid CDx were performed on tissue and liquid biopsies, respectively, according to methods previously described (5–7). Both tissue- and liquid-based testing assessed 324 cancer-related genes, including select intronic/other regions. In this study, CGP results were analyzed for short variants, including single-base substitutions and short insertions and deletions. Genomic ancestry of patients was determined using analysis of genomic SNPs trained on data from the 1000 Genomes Project (RRID: SCR_006828), with each patient classified as belonging to one of the following subpopulations: African, East Asian, European, South Asian, or admixed American (8, 9).

### ClinVar database analysis

ClinVar (RRID: SCR_006169) data pulls from March 13th, 2022, and March 1st, 2024, were analyzed (https://ftp.ncbi.nlm.nih.gov/pub/clinvar/vcf_GRCh37/). Clinical significance was summarized as pathogenic/likely pathogenic (P/LP), benign/likely benign (B/LB), or VUS according to the level of evidence in ClinVar (Supplementary Table S2). In brief, the level of evidence required for P/LP was ClinVar classification as P/LP by ≥2 submitters or by an expert panel, and for B/LB was ClinVar classification as B/LB by multiple submitters without conflicts or by an expert panel. All other variants were considered VUS.

### Statistical analysis

Statistical tests were performed using Python (RRID: SCR_008394; v3.9.12). Fisher exact tests were carried out using the “fisher_exact” function from the statistical functions module (scipy.stats) of SciPy (RRID: SCR_008058; v1.7.3), and corrections for multiple hypothesis tests were performed using the Benjamini–Hochberg procedure with the “fdr_bh” method for the “multipletests” function from the statsmodels (v0.13.2) Python package.

### Data availability

The authors declare that all relevant aggregate data supporting the findings of this study are available within the article and its supplementary information files. The data that support the findings of this study originated from Foundation Medicine, Inc. In accordance with the Health Insurance Portability and Accountability Act, we do not have Institutional Review Board approval or patient consent to share individualized patient genomic data, which contains potentially identifying or sensitive patient information and cannot be reported in a public data repository. Foundation Medicine is committed to collaborative data analysis and has well established and widely used mechanisms by which qualified researchers can query our core genomic database of >900,000 deidentified sequenced cancers. Interested academic researchers can submit a proposal to the Foundation Medicine Data Collaborations Committee. More information and mechanisms for data access can be obtained by contacting the corresponding author or the Foundation Medicine Data Governance Council at data.governance.council@foundationmedicine.com.

## Results

### Impact of ClinVar database evolution on available evidence for PPGV classification

**Figure 1:**
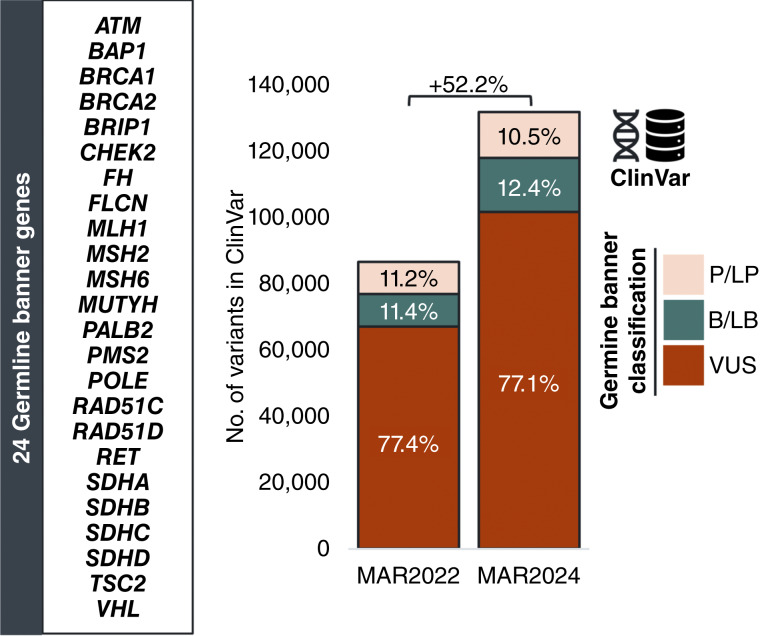
Evolution of the ClinVar database with respect to the 24 germline banner genes. Germline banner classification of short variants (single-nucleotide variants and short insertions/deletions) in the 24 germline banner genes using ClinVar evidence (see “Materials and Methods”, Supplementary Table S2) from March 2022 vs. March 2024. The proportions of P/LP, B/LB, and VUS variants in the database at each time point are indicated. B/LB, benign/likely benign; P/LP, pathogenic/likely pathogenic; VUS, variant of uncertain significance.

We compared the number of short variants (single-nucleotide variants or short insertions/deletions) classified in the ClinVar database within the 24 germline banner genes in March 2022 versus March 2024. Overall, the number of ClinVar germline variants in the database—inclusive of P/LP, B/LB, and VUS variants using the classification criteria for germline banner reporting (See “Materials and Methods”, Supplementary Table S2)—increased by 52.2% (+45,174 variants). The number of variants with sufficient evidence to be classified as P/LP—ClinVar classification as P/LP by ≥2 submitters or by an expert panel—increased by 42.7% (+4,132), whereas the number of B/LB increased by 65.0% (+6,427) and the number of VUS by 51.6% (+34,615). The relative proportions of P/LP, B/LB, and VUS variants in the database remained similar from year to year (11.2% vs. 10.5% P/LP, 11.4% vs. 12.4% B/LB, and 77.4% vs. 77.1% VUS; Fig. 1). The changes in available evidence varied by gene (Supplementary Fig. S1).

### Impact of ClinVar database evolution on germline banner reporting of PPGVs

**Figure 2:**
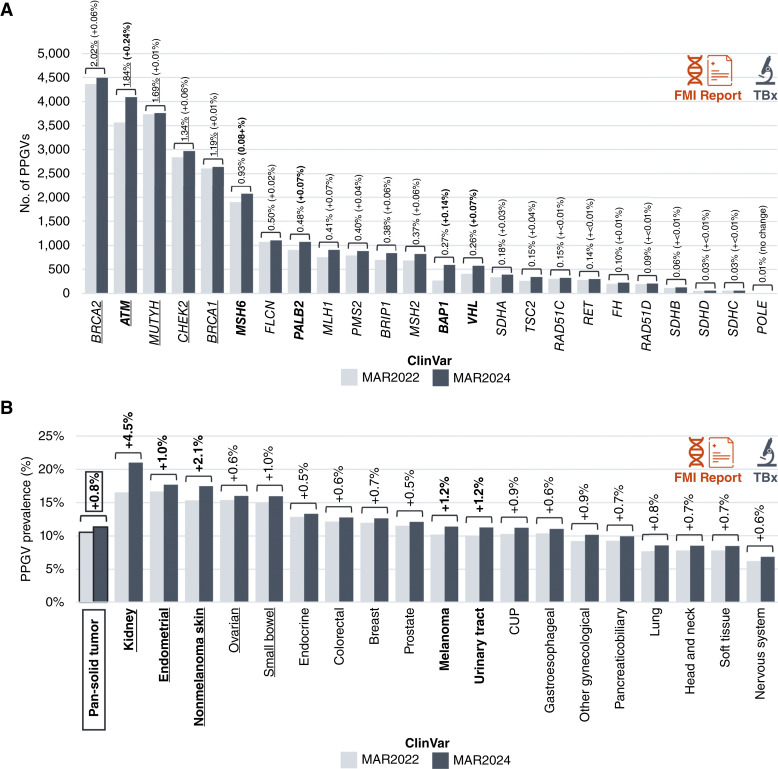
Impact of ClinVar database evolution on germline banner reporting of PPGVs in TBx (*N* = 221,241). **A,** Number of PPGVs reported across the 24 germline banner genes in TBx using ClinVar evidence from March 2022 vs. March 2024. The overall prevalence of PPGVs in the cohort in March 2024 and the % change in PPGV prevalence between March 2022 and March 2024 (in parentheses) for each gene is indicated. The top five genes with the highest PPGV prevalence in March 2024 are underlined, and those with the highest percentage increase in PPGV prevalence over the 2-year period are in bold. **B,** Prevalence of PPGV+ TBx cases across cancer types using ClinVar evidence from March 2022 vs. March 2024. The % change in prevalence between March 2022 and March 2024 in each cancer type is indicated. The top five cancer types with the highest PPGV prevalence in March 2024 are underlined, and those with the highest percentage increase in PPGV prevalence over the 2-year period are in bold. Only cancer types with ≥100 PPGV+ TBx cases in March 2024 are shown. CUP, unknown primary carcinoma; FMI, Foundation Medicine, Inc.; PPGV, potential pathogenic germline variant; TBx, tissue biopsy.

We identified PPGVs in a pan-solid tumor cohort of 289,547 patients who received either tissue (*N* = 222,241) or liquid (*N* = 67,306) CGP between January 2021, following launch of the germline banner on Foundation Medicine reports, and July 2024 using ClinVar evidence from the two time points (March 2022 vs. March 2024; Supplementary Table S1). The method for classifying PPGVs highlighted in the germline banner has been described in detail previously (2). Briefly, the criteria restrict to short variants that are (i) detected in 24 cancer susceptibility genes (*ATM*, *BAP1*, *BRCA1*, *BRCA2*, *BRIP1*, *CHEK2*, *FH*, *FLCN*, *MLH1*, *MSH2*, *MSH6*, *MUTYH*, *PALB2*, *PMS2*, *POLE*, *RAD51C*, *RAD51D*, *RET*, *SDHA*, *SDHB*, *SDHC*, *SDHD*, *TSC2*, and *VHL*), (ii) have a variant allele fraction (VAF) above designated thresholds for tissue CGP (≥10%) or liquid CGP (≥30%), respectively, and (iii) are classified as germline P/LP in ClinVar with a sufficient level of evidence (See “Materials and Methods”, Supplementary Table S2). The overall number of PPGVs identified in tissue biopsies increased by 7.7% (+1,809) using March 2022 versus March 2024 ClinVar data. Genes with the greatest numeric increase in PPGVs were *BAP1* (+526 PPGV), *SDHA* (+321), *MLH1* (+172), *MSH2* (+166), and *FLCN* (+164). Genes with the greatest percentage increase in PPGVs were *BAP1* (+118.5% PPGV), *VHL* (+39.8%), *TSC2* (+30.3%), *MLH1* (+20.0%), and *BRIP1* (+19.9%; Fig. 2A). PPGV prevalence in tissue biopsies across solid tumor types increased by 0.8% (10.6% increasing to 11.4%; range, +0.5% to +4.5% across cancer types), with the greatest percentage increases in kidney (+4.5%), nonmelanoma skin (+2.1%), urinary tract (+1.2%), melanoma (+1.2%), and endometrial (+1.0%) cancers (Fig. 2B; Supplementary Fig. S2).

The overall number of PPGVs identified in liquid biopsies increased by 2.5% (+116) using March 2022 versus March 2024 ClinVar data. Genes with the greatest numeric increase in PPGVs were *ATM* (+38 PPGV), *PALB2* (+12), *BRIP1* (+12), *MSH6* (+10), and *SDHA* (+10). Genes with the greatest percentage increase in PPGVs were *VHL* (+40.0% PPGV), *TSC2* (+18.2%), *SDHA* (+12.5%), *SDHB* (+11.8%), and *MSH6* (+10.6%; Supplementary Fig. S3A). PPGV prevalence in liquid biopsies across solid tumor types increased by 0.2% (7.0% increasing to 7.1%; range, −0.2% to +0.3% across cancer types), with the greatest percentage increases in kidney (+0.3%), gastroesophageal (+0.3%), breast (+0.3%), pancreaticobiliary (+0.3%), and unknown primary carcinoma (+0.2%) cancers (Supplementary Fig. S3B). Gene-specific PPGV prevalence across cancer types in both tissue and liquid biopsies based on March 2024 ClinVar evidence is presented in Supplementary Fig. S4.

We observed that significant increases in the number of classified variants in the ClinVar database led to relatively modest increases in identified PPGVs for most genes (Supplementary Fig. S5A). For example, a 26.3% increase in *BRCA1/2* ClinVar entries resulted in a 6.8% increase in P/LP variants that had a sufficient level of evidence for germline banner reporting, which converted to only a 2.3% increase in PPGVs identified in tissue biopsies (and a 0.7% increase in liquid biopsies). We therefore assessed the ClinVar evidence available for all novel *BRCA1/2* variants (i.e., *BRCA* variants found in ClinVar in the March 2024 data pull but not in the March 2022 data pull; *N* = 6,683). A significant proportion of novel *BRCA1/2* entries were classified as either VUS (31.6%) or B/LB (25.6%) in ClinVar, with 42.8% classified as P/LP. Moreover, only 6.7% of novel P/LP variant entries were supported by classification by multiple submitters (6.7%) or an expert panel (<0.1%), with 89.2% of entries supported by evidence from only a single submitter and 4.1% with no assertion criteria provided (Supplementary Fig. S5B).

To understand the impact of ClinVar database evolution on PPGV filtering, we looked more closely at germline banner gene variants present in tumor at the required VAF which were filtered out (i.e., not classified as PPGV) based on the level of evidence in the ClinVar database. Using March 2024 ClinVar evidence reduced the number of ClinVar-filtered variants in tissue biopsies by 14.6% compared with when using March 2022 evidence (−2672 variants; Supplementary Fig. S6). The proportion of variants excluded because they were not classified in ClinVar decreased from 60.7% to 55.1%, reflecting an overall increase in available evidence within the ClinVar database.

**Figure 3:**
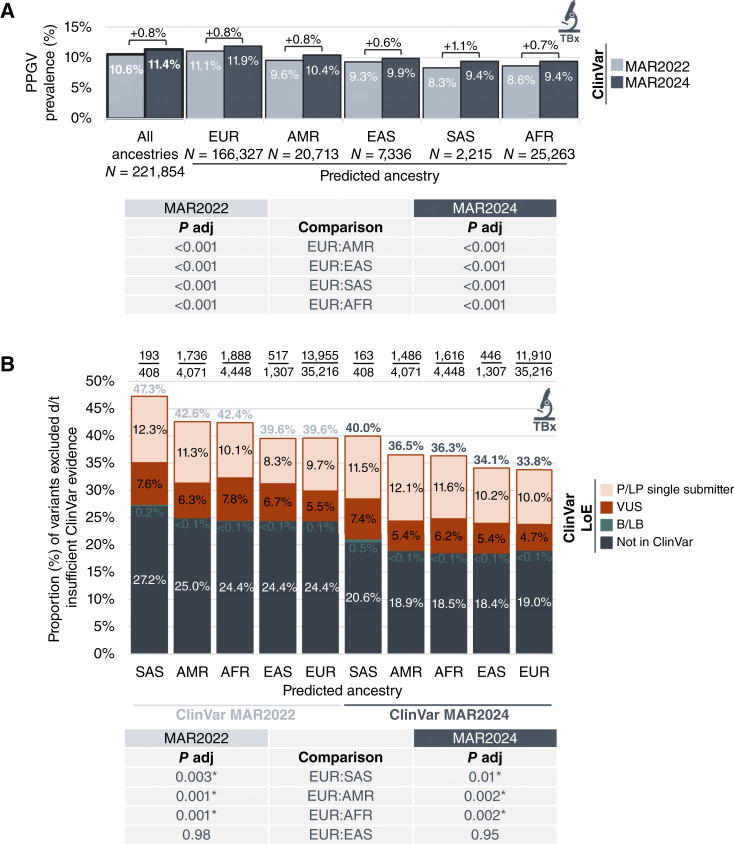
Impact of ClinVar database evolution on PPGV classification by ancestry. **A,** PPGV prevalence in TBx across genomic ancestries based on ClinVar evidence from March 2022 vs. March 2024. Only samples with available genomic ancestry calls were included (*N* = 221,854, >99% of the cohort). **B,** The relative proportions of variants with sufficient VAF excluded as PPGVs based on insufficient ClinVar evidence from March 2022 vs. March 2024 across genomic ancestries. Genomic ancestry reflects the 1000 Genomes Project superpopulations: AFR, African; AMR, Admixed American; EAS, East Asian; EUR, European; and SAS, South Asian. Statistical analysis was performed using the Fisher exact test with the Benjamini–Hochberg procedure for multiplicity correction [adjusted *P* value (*P* adj)]. Asterisk (*) indicates a statistically significant difference (*P* < 0.05). B/LB, benign/likely benign; LoE; level of evidence; P/LP, pathogenic/likely pathogenic; PPGV, potential pathogenic germline variant; TBx, tissue biopsy; VUS, variant of uncertain significance.

We also investigated whether ClinVar database evolution lessened disparities previously observed in PPGV variant classification and filtering (2). Whereas PPGV prevalence in tissue biopsies increased across all ancestry groups using evidence from March 2024 versus March 2022 (range, +0.6% to +1.1%), significantly higher PPGV prevalence was still seen in European versus non-European populations [11.9% European vs. 10.4% admixed American, 9.9% East Asian, 9.4% South Asian, and 9.4% African (*P* <0.001 for all comparisons); Fig. 3A]. The proportion of ClinVar-filtered variants decreased across all ancestry groups (South Asian −7.3%; admixed American −6.1%; African −6.1%; East Asian −5.5%; and European −5.8%), but there was still a significantly higher percentage of variants filtered out in patients of South Asian (40.0%, *P* = 0.01), admixed American (36.5%, *P* = 0.002), and African (36.3%, *P* = 0.002) versus European (33.8%) ancestry (Fig. 3B). This was attributable to higher proportions of both VUS (South Asian 7.4%, admixed American 5.4%, and African 6.2% vs. European 4.7%) and P/LP variants with an insufficient level of evidence for germline banner reporting (South Asian 11.5%, admixed American 12.1%, and African 11.6% vs. European 10.0%), reflecting improved but continuing underrepresentation of these populations in the ClinVar database.

## Discussion

Discovery of PPGVs through tumor CGP is an important opportunity to identify patients and families with high inherited cancer risk; therefore, optimizing the criteria for PPGV identification is essential. We evaluated the impact of ClinVar database evolution from March 2022 to March 2024 on PPGV classification across the 24 cancer susceptibility genes included in the Foundation Medicine germline banner. Substantial growth in the ClinVar database—a 52% increase in classified variants over 2 years—yielded a modest increase in PPGV prevalence (0.8% in tissue and 0.2% in liquid biopsy specimens). This discrepancy may be due to an abundance of VUS in the ClinVar database and the fact that most new P/LP classifications relied upon evidence from a single submitter, thus failing to meet the evidence threshold for PPGV classification, as well as the rarity of specific germline variants in the population.

Our evidence threshold for PPGV classification—namely a variant detected in 1 of 24 cancer susceptibility genes with a sufficient VAF and classified as germline P/LP in ClinVar with a sufficient level of evidence—significantly influenced the study results. A strict threshold was selected to improve specificity, with the aim of moderating germline testing rates. This approach aligns with general principles of population screening for germline genetic testing during the first two decades of its use, when costs were high and testing guidelines were parsimonious (10). However, germline testing guidelines have expanded more recently, reflecting a higher than anticipated prevalence of pathogenic germline variants in many common cancer types and the growing relevance of pathogenic germline variants for cancer therapy (11–13). Current practice guidelines recommend testing up to 80% of patients with female breast cancer, nearly all patients with ovarian and pancreatic cancers, and substantial proportions of patients with colorectal, prostate, and endometrial cancers (14, 15). Notably, we also observed a high prevalence of PPGVs in several cancer types that are not indicated for universal germline testing, including kidney, nonmelanoma skin, and small bowel cancers. Accordingly, a more liberal threshold for PPGV classification—e.g., allowing ClinVar P/LP classification by a single submitter to be sufficient evidence—might be considered, with the potential to address underutilization of germline testing by recommending germline follow-up testing after CGP for a greater percentage of patients (1). Furthermore, results from exploring classification data shared in ClinVar reflect the historic rarity of germline testing as evidenced by a high volume of VUS/conflicting interpretations of pathogenicity and many P/LP germline variant classifications that are based on data from a single institution/laboratory. Both wider adoption of germline testing and increased data sharing should improve these limitations.

A key finding was the persistence of racial and ethnic disparities in PPGV filtering even with ClinVar database evolution over time. Although an increase in PPGV prevalence was observed across all genomic ancestry groups due to the increased volume of shared classification data in ClinVar from year-to-year, prevalence was still significantly higher in European versus non-European populations; this disparity arose both from a higher prevalence of VUS in minoritized groups and from their overall underrepresentation in ClinVar. We and others have noted the greater burden of VUS in racial and ethnic minoritized groups, a gap that has widened with larger gene sequencing panels in clinical practice (1, 4, 16). Whereas the observed increase in ClinVar data did improve disparities that we previously reported (2), the current evolution was not sufficient to close the still salient racial and ethnic gap in testing results. Efforts to overcome barriers to accessing genetic testing for these populations, as well as ongoing data sharing to improve alignment on classification of population-specific variants (including rare founder variants), will be essential to eradicate persistent racial and ethnic disparities (17–20).

In summary, we found that increases in data sharing to ClinVar over time translated into a modest increase in PPGV classification from CGP of tissue and liquid biopsy specimens and noteworthy improvement of racial and ethnic disparities in classifying PPGVs. A multifaceted approach that involves addressing barriers to genetic testing access and facilitating ongoing data sharing to improve variant classification is needed to make meaningful progress.

## Supplementary Material

Supplementary Figure S1 Evolution Of The ClinVar Database With Respect To The 24 Germline Banner Genes – Gene Level

Supplementary Figure S2 Impact Of ClinVar Database Evolution On PPGV Reporting Across Cancer Types

Supplementary Figure S3 Impact Of ClinVar Database Evolution On Germline Banner Reporting Of PPGVs In LBx (N = 67,306)

Supplementary Figure S4 Potential Pathogenic Germline Variant (PPGV) Pan-Solid Tumor Landscape (March 2024)

Supplementary Figure S5 Variant Exclusion Based On Insufficient ClinVar Evidence

Supplementary Figure S6 Conversion Of Evolving ClinVar Evidence To Newly Identified PPGVs

Supplementary Table S1 Pan-Solid Tumor Comprehensive Genomic Profiling (CGP) Cohort Cancer Types

Supplementary Table S2 ClinVar Levels Of Evidence For Germline Banner Classification
