# Supplementary figures and images for: ClinVar Database Evolution and Impact on Potential Pathogenic Germline Variant Reporting from Tumor Comprehensive Genomic Profiling
Source: Cancer Res Commun. 2025 Aug 5;5(8):1282–7. doi: 10.1158/2767-9764.CRC-25-0038 (PMC12322967; doi:10.1158/2767-9764.CRC-25-0038)

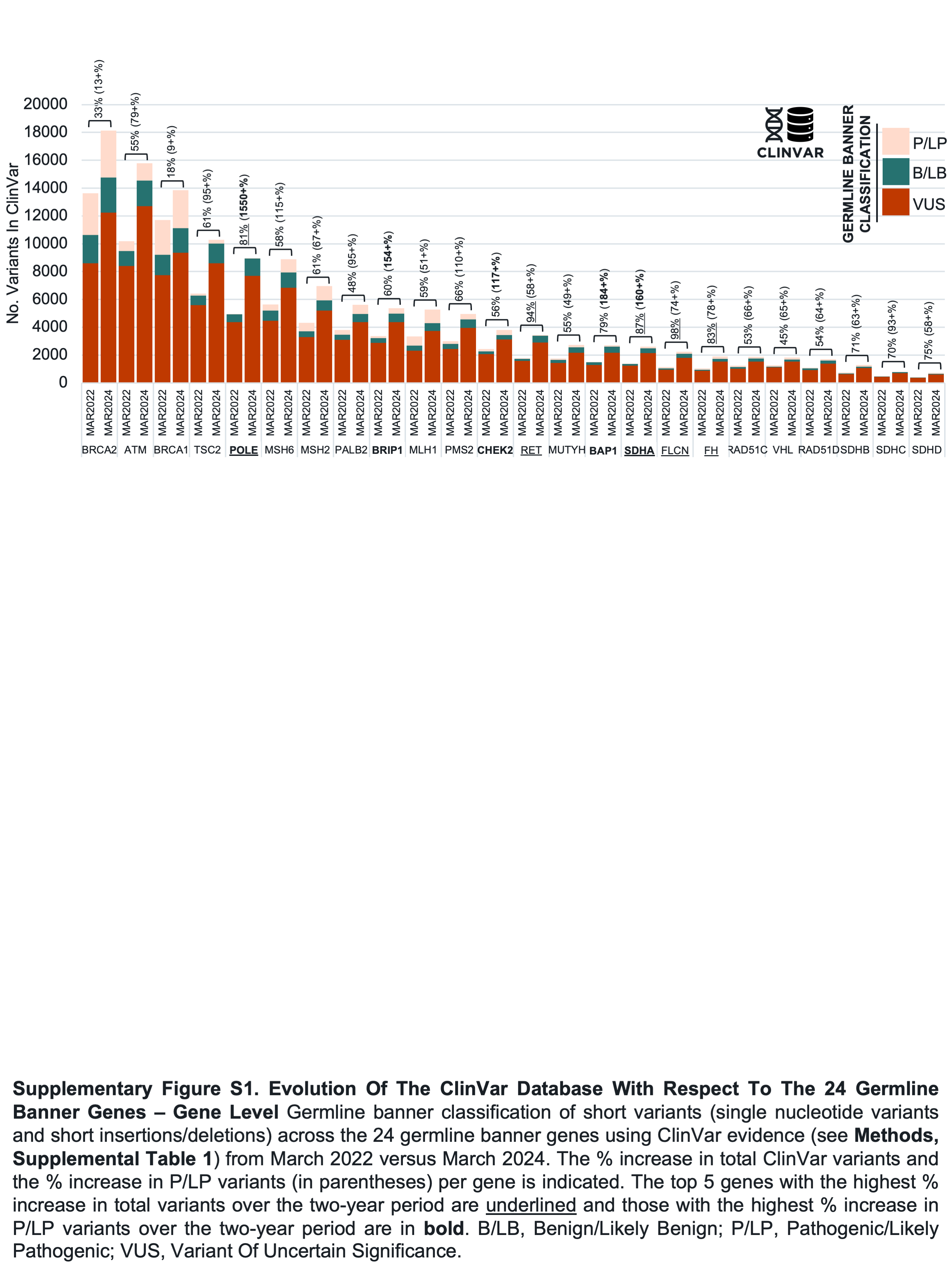

Supplement: Supplementary Figure S1 — Evolution Of The ClinVar Database With Respect To The 24 Germline Banner Genes – Gene Level [file crc-25-0038_supplementary_figure_s1_suppsf1.png]

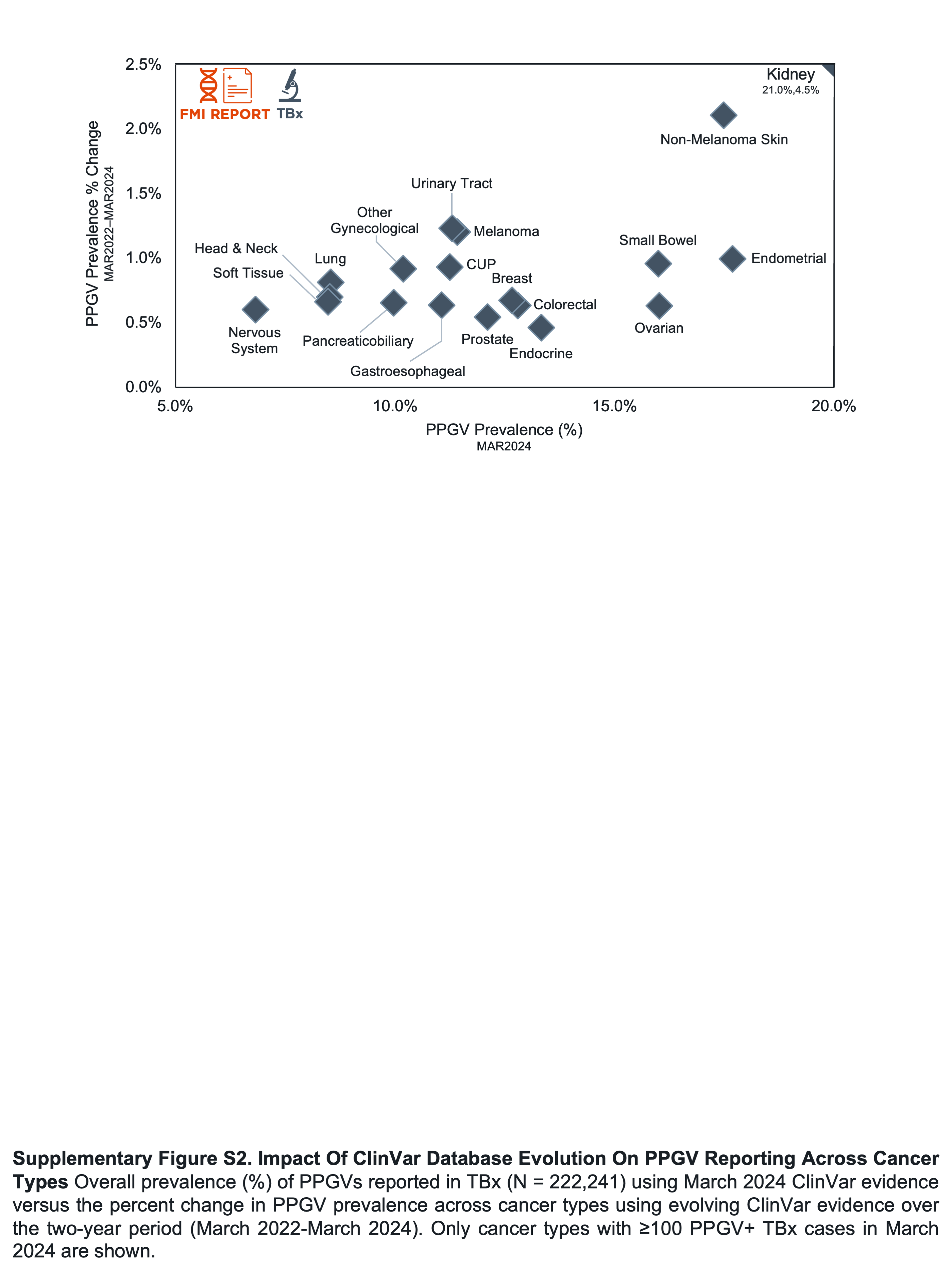

Supplement: Supplementary Figure S2 — Impact Of ClinVar Database Evolution On PPGV Reporting Across Cancer Types [file crc-25-0038_supplementary_figure_s2_suppsf2.png]

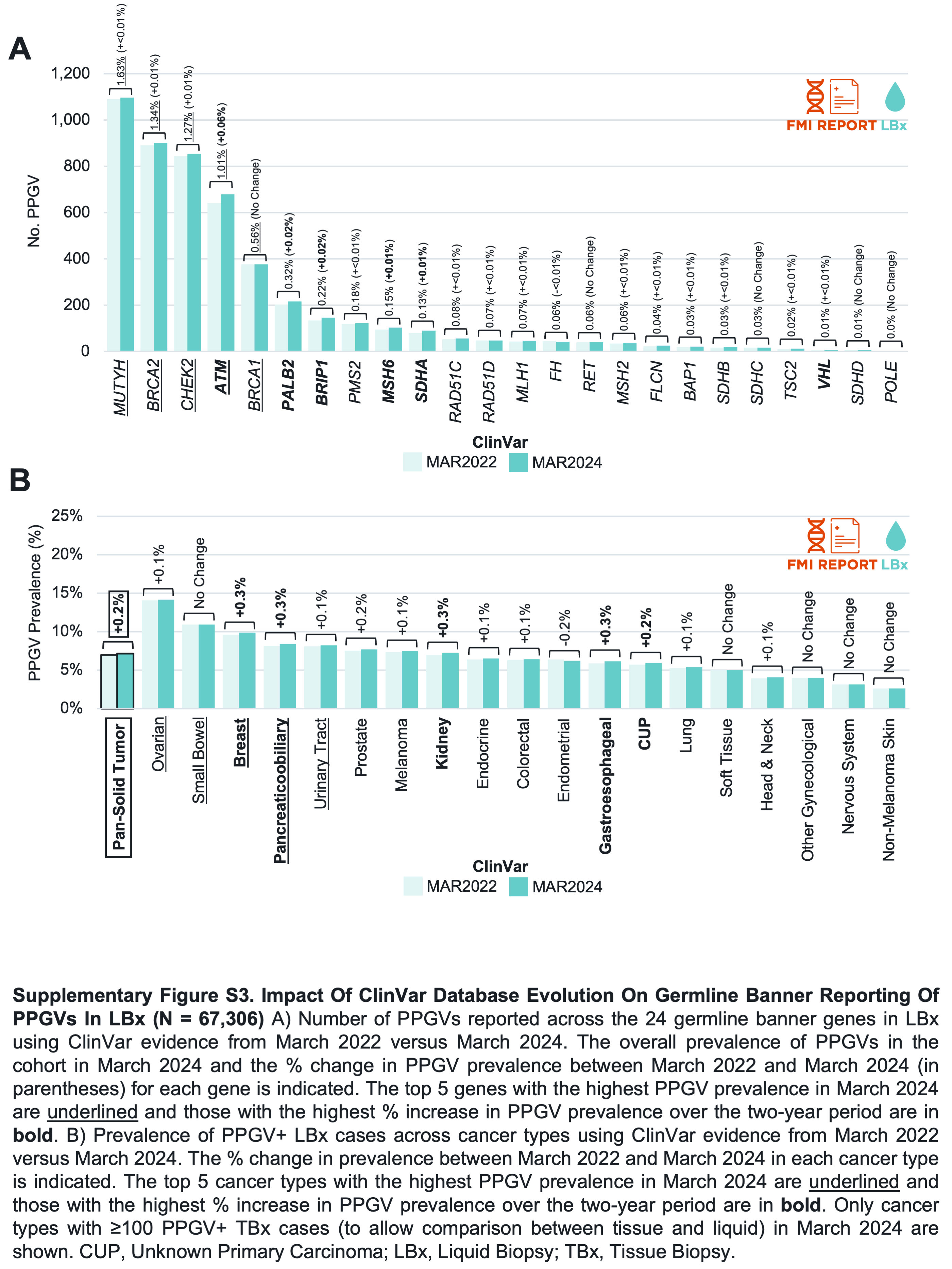

Supplement: Supplementary Figure S3 — Impact Of ClinVar Database Evolution On Germline Banner Reporting Of PPGVs In LBx (N = 67,306) [file crc-25-0038_supplementary_figure_s3_suppsf3.png]

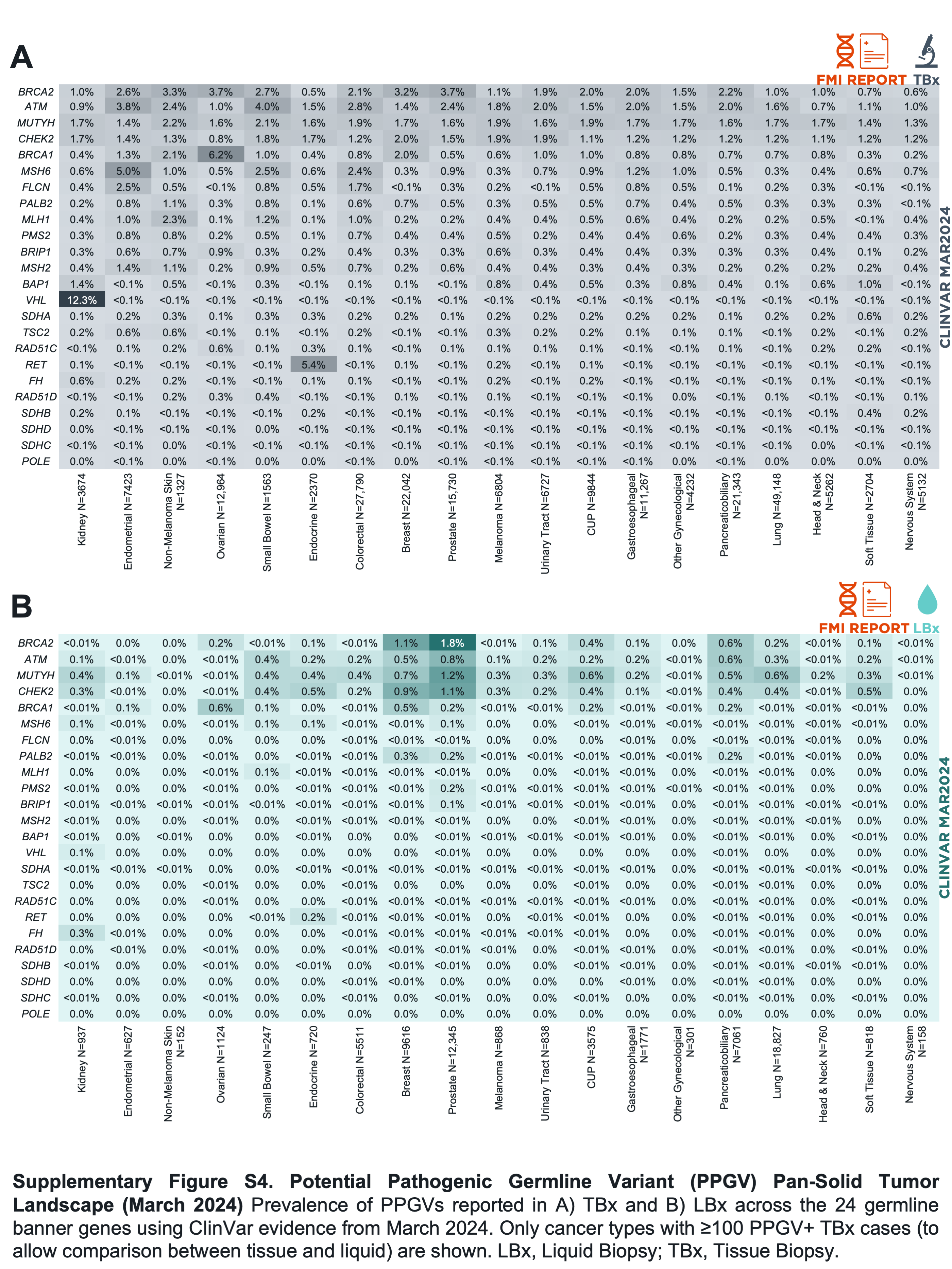

Supplement: Supplementary Figure S4 — Potential Pathogenic Germline Variant (PPGV) Pan-Solid Tumor Landscape (March 2024) [file crc-25-0038_supplementary_figure_s4_suppsf4.png]

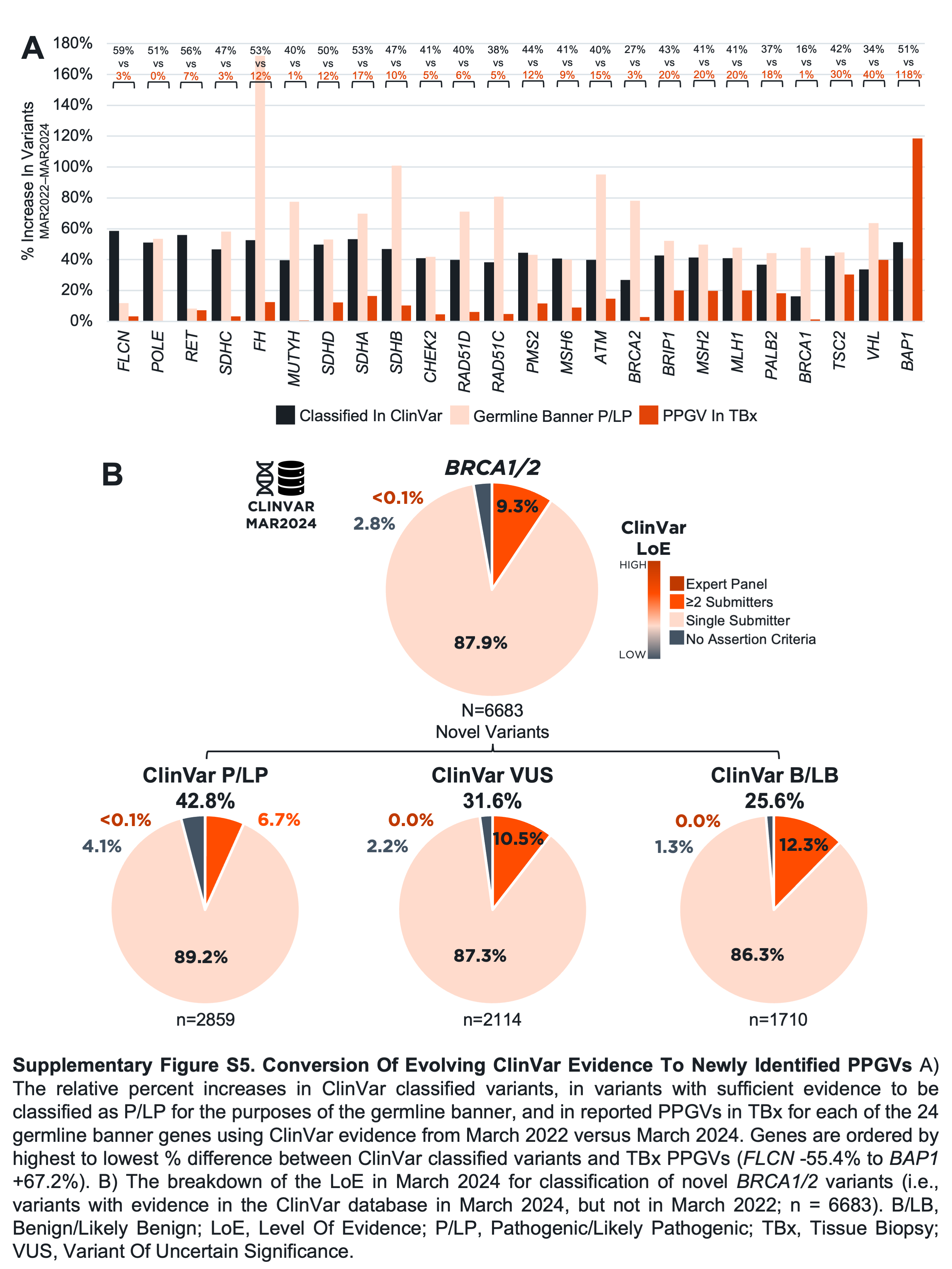

Supplement: Supplementary Figure S5 — Variant Exclusion Based On Insufficient ClinVar Evidence [file crc-25-0038_supplementary_figure_s5_suppsf5.png]

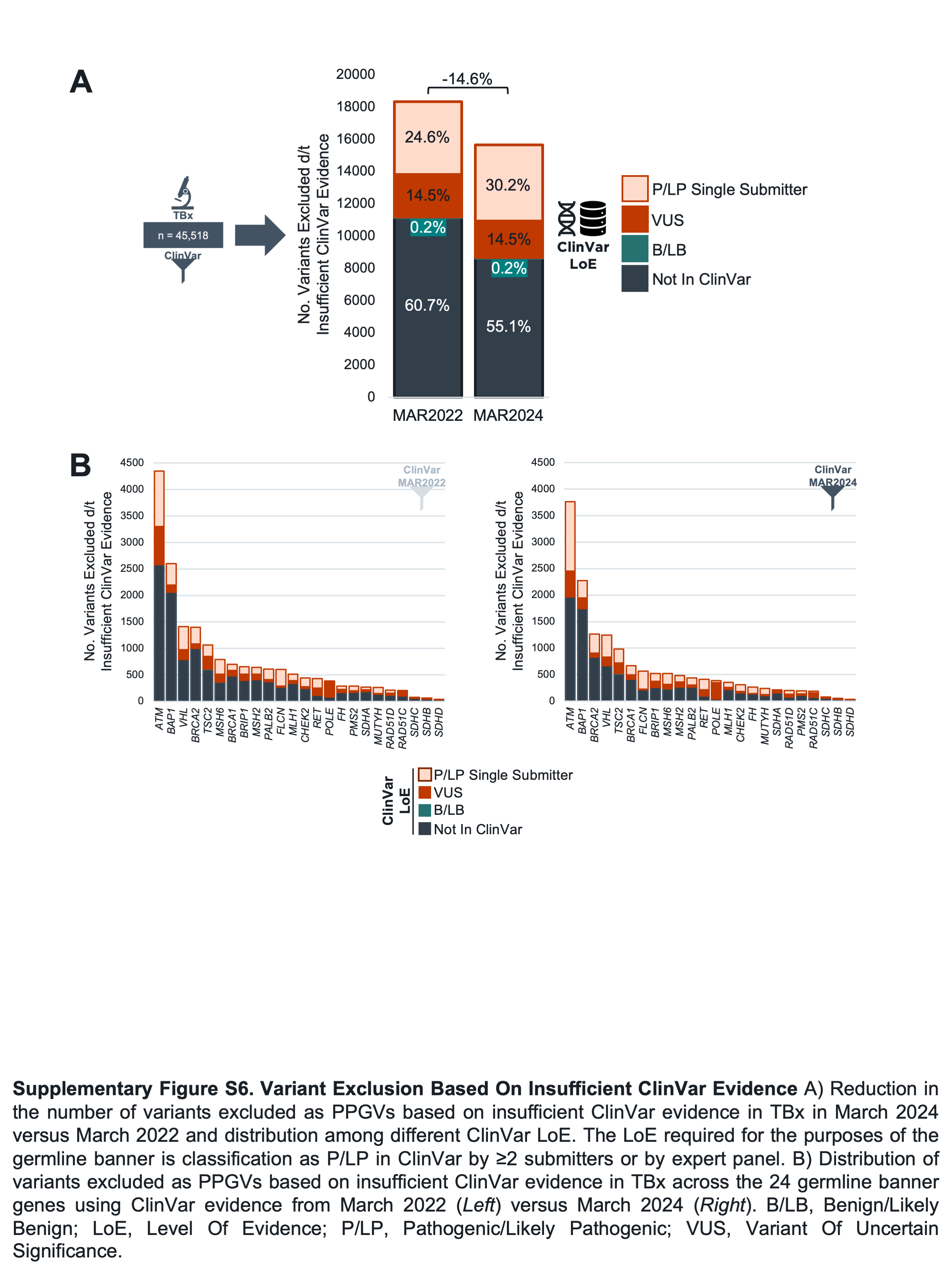

Supplement: Supplementary Figure S6 — Conversion Of Evolving ClinVar Evidence To Newly Identified PPGVs [file crc-25-0038_supplementary_figure_s6_suppsf6.png]

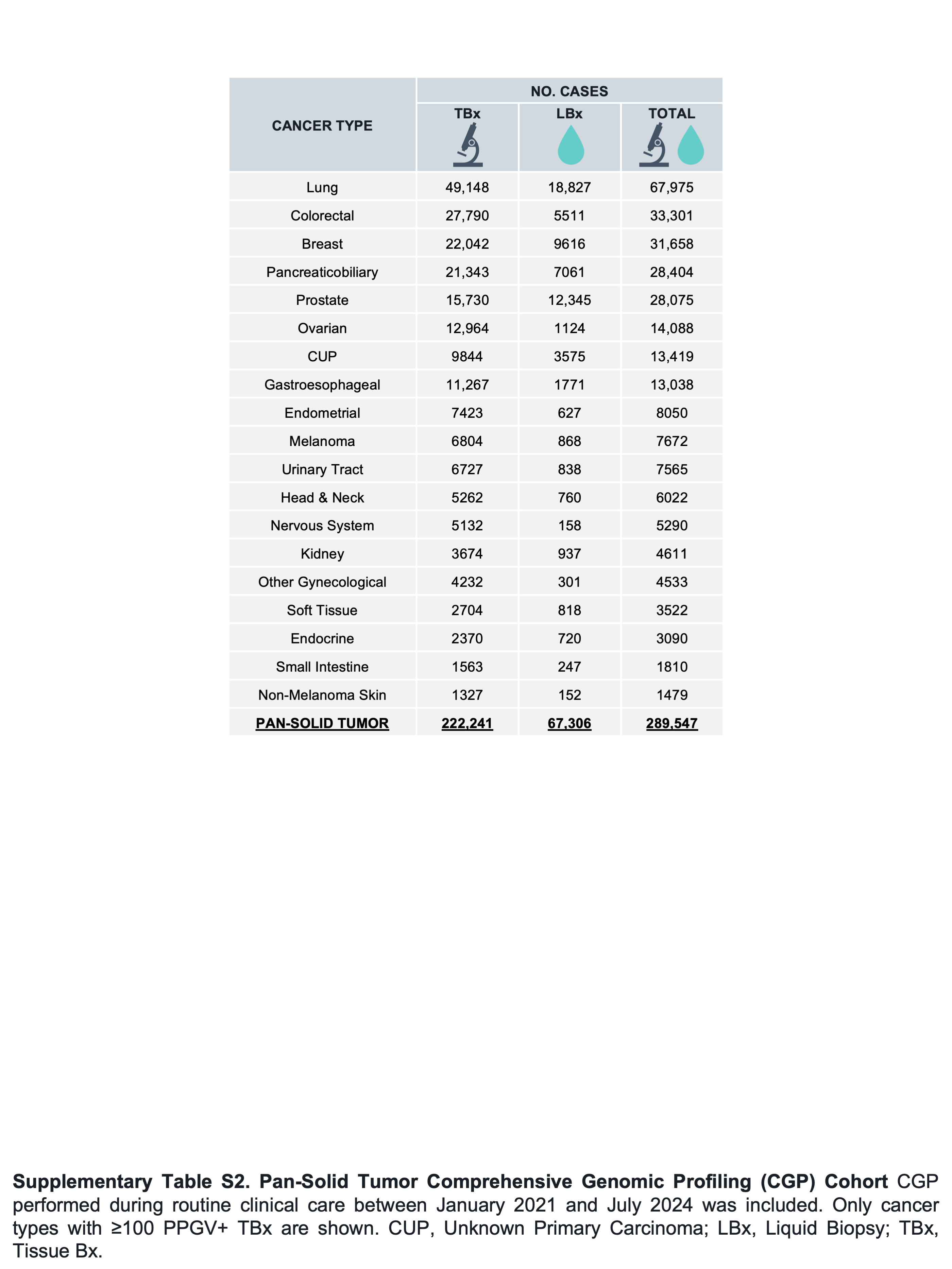

Supplement: Supplementary Table S1 — Pan-Solid Tumor Comprehensive Genomic Profiling (CGP) Cohort Cancer Types [file crc-25-0038_supplementary_table_s1_suppst1.png]

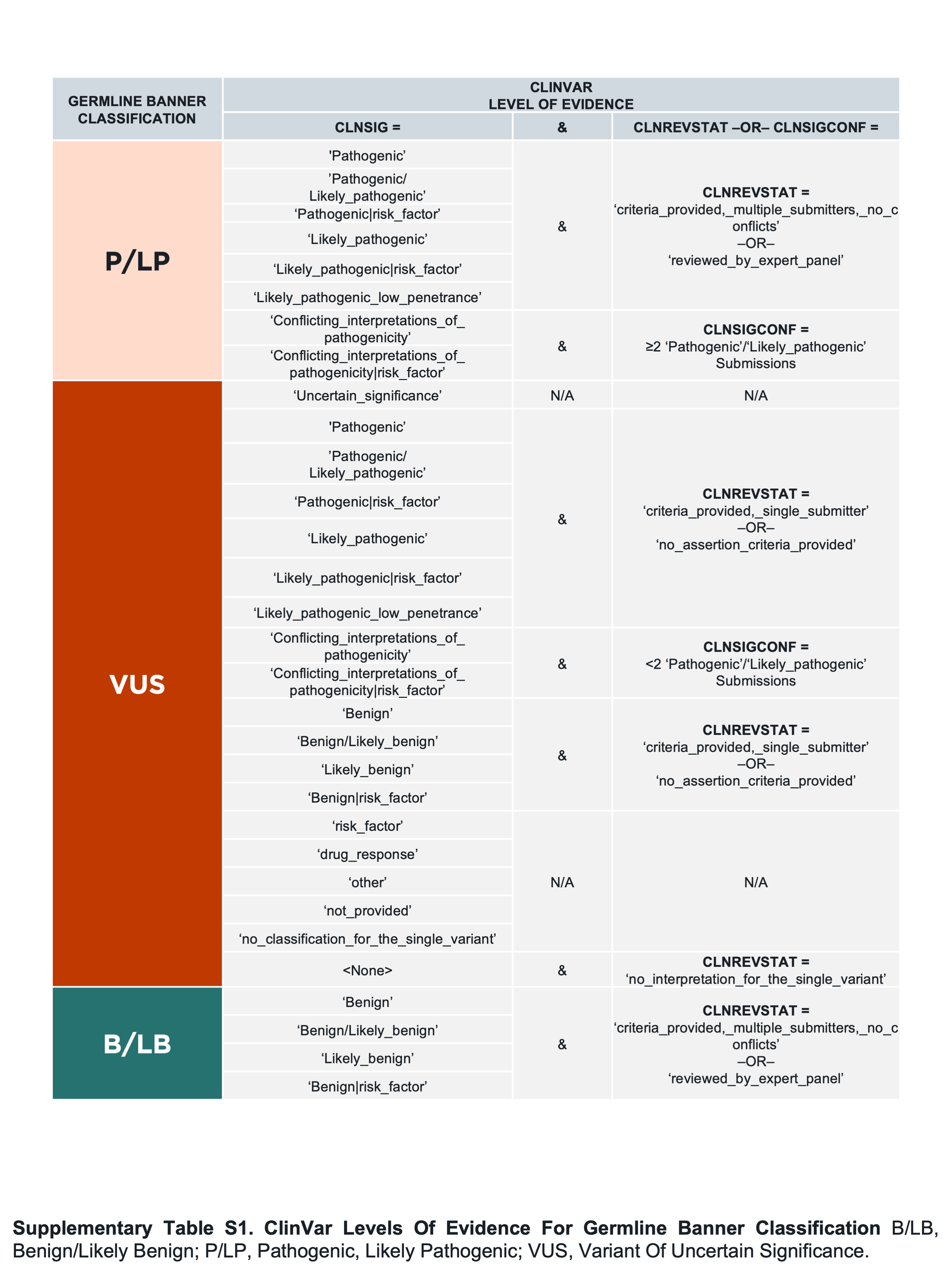

Supplement: Supplementary Table S2 — ClinVar Levels Of Evidence For Germline Banner Classification [file crc-25-0038_supplementary_table_s2_suppst2.png]
